# Supplementary material for: Multidimensional performance assessment, government competition and ecological welfare performance
Source: PLoS One. 2023 Aug 10;18(8):e0289837. doi: 10.1371/journal.pone.0289837 (PMC10414661; doi:10.1371/journal.pone.0289837)
Supplement: S1 Appendix — (DOCX) [file pone.0289837.s001.docx]

# Appendix

**Table 10** The short-term and long-term effect under multidimensional assessment systems (test)

| Period | Test | 1-9 | 2-8 | 3-7 | 4-6 | 5-5 | 6-4 | 7-3 | 8-2 | 9-1 |
| --- | --- | --- | --- | --- | --- | --- | --- | --- | --- | --- |
| Current year | AR(1) | -2.25 | -2.21 | -2.23 | -2.23 | -2.23 | -2.23 | -2.22 | -2.22 | -2.21 |
|  |  | [0.025] | [0.027] | [0.026] | [0.026] | [0.026] | [0.026] | [0.027] | [0.026] | [0.027] |
|  | AR(2) | 1.49 | 1.48 | 1.48 | 1.46 | 1.45 | 1.44 | 1.42 | 1.41 | 1.40 |
|  |  | [0.135] | [0.139] | [0.140] | [0.143] | [0.147] | [0.151] | [0.156] | [0.157] | [0.160] |
|  | Hansen test | 21.99 | 22.48 | 26.2 | 25.8 | 25.21 | 24.74 | 26.29 | 24.95 | 24.9 |
|  |  | [0.928] | [ 0.916] | [0.794] | [0.810] | [0.832] | [0.849] | [ 0.790] | [0.842] | [0.844] |
| Future 1 | AR(1) | -2.18 | -2.18 | -2.19 | -2.21 | -2.22 | -2.23 | -2.23 | -2.26 | -2.27 |
|  |  | [ 0.029] | [0.029] | [0.028] | [0.027] | [0.027] | [0.026] | [0.025] | [0.024] | [0.023] |
|  | AR(2) | 1.31 | 1.33 | 1.37 | 1.4 | 1.42 | 1.43 | 1.43 | 1.44 | 1.44 |
|  |  | [0.189] | [0.183] | [0.171] | [0.162] | [0.155] | [0.153] | [0.153] | [1.44] | [0.150] |
|  | Hansen test | 26.43 | 27.13 | 26.7 | 26.39 | 25.02 | 26.87 | 26.67 | 25.03 | 24.49 |
|  |  | [0.784] | [0.754] | [0.772] | [0.786] | [0.839] | [0.765] | [0.774] | [0.839] | [0.858] |
| Future 2 | AR(1) | -2.28 | -2.28 | -2.28 | -2.28 | -2.28 | -2.27 | -2.27 | -2.27 | -2.30 |
|  |  | [0.023] | [0.023] | [0.022] | [0.023] | [0.022] | [0.023] | [0.023] | [0.023] | [0.022] |
|  | AR(2) | 1.47 | 1.46 | 1.46 | 1.45 | 1.44 | 1.42 | 1.41 | 1.41 | 1.43 |
|  |  | [0.142] | [0.145] | [0.145] | [0.148] | [0.150] | [0.155] | [0.158] | [0.158] | [0.152] |
|  | Hansen test | 26.00 | 26.88 | 26.42 | 26.09 | 25.02 | 24.98 | 25.08 | 24.78 | 20.03 |
|  |  | [0.802] | [0.765] | [0.785] | [0.798] | [0.839] | [0.841] | [0.837] | [0.848] | [0.963] |
| Future 3 | AR(1) | -2.34 | -2.36 | -2.32 | -2.34 | -2.40 | -2.41 | -2.38 | -2.38 | -2.37 |
|  |  | [0.019] | [0.018] | [0.020] | [0.019] | [0.016] | [0.016] | [0.017] | [0.017] | [0.018] |
|  | AR(2) | 1.38 | 1.39 | 1.38 | 1.40 | 1.43 | 1.44 | 1.43 | 1.43 | 1.42 |
|  |  | [0.168] | [0.165] | [0.166] | [0.161] | [0.152] | [0.149] | [0.152] | [0.152] | [0.155] |
|  | Hansen test | 24.97 | 23.24 | 25.63 | 26.16 | 24.65 | 23.43 | 23.94 | 23.96 | 24.24 |
|  |  | [0.807] | [0.871] | [0.780] | [0.757] | [0.820] | [0.864] | [0.847] | [0.846] | [0.835] |
| Future 4 | AR(1) | -2.20 | -2.20 | -2.17 | -2.16 | -2.15 | -2.12 | -2.16 | -2.20 | -2.21 |
|  |  | [0.028] | [0.028] | [0.030] | [0.031] | [0.031] | [0.034] | [0.031] | [ 0.028] | [0.027] |
|  | AR(2) | 1.23 | 1.24 | 1.24 | 1.25 | 1.26 | 1.26 | 1.26 | 1.26 | 1.26 |
|  |  | [0.220] | [0.217] | [0.217] | [0.211] | [0.208] | [0.207] | [0.209] | [0.206] | [0.206] |
|  | Hansen test | 24.72 | 25.27 | 26.68 | 25.36 | 24.13 | 23.27 | 24.05 | 23.12 | 22.69 |
|  |  | [0.693] | [0.664] | [0.589] | [0.660] | [0.722] | [0.764] | [0.726] | [0.771] | [0.791] |
